# Combination of *Bacillus licheniformis* and Salinomycin: Effect on the Growth Performance and GIT Microbial Populations of Broiler Chickens

**DOI:** 10.3390/ani10050889

**Published:** 2020-05-20

**Authors:** Jacek Trela, Bartosz Kierończyk, Veerle Hautekiet, Damian Józefiak

**Affiliations:** 1 Huvepharma Poland Sp. z.o.o., ul. Aleje Jerozolimskie 146D, 02-305 Warsaw, Poland; jacek.trela@huvepharma.com; 2 Department of Animal Nutrition, Poznań University of Life Sciences, Wołyńska 33, 60-637 Poznań, Poland; damian.jozefiak@up.poznan.pl; 3 Huvepharma NV, Uitbreidingstraat 80, 2600 Antwerp, Belgium; veerle.hautekiet@huvepharma.com

**Keywords:** feed additive, probiotic, *Bacillus licheniformis*, ionophore coccidiostat, salinomycin, broiler chicken, performance, microbiota

## Abstract

**Simple Summary:**

The beneficial effects of *Bacillus* spp. probiotic preparations used for poultry are well-documented and characterized by growth performance improvement and positive modulation of gastrointestinal tract (GIT) microbiota. Moreover, the favorable influence of salinomycin has been frequently studied as an ionophore coccidiostat, as well as an antimicrobial agent. However, limited data are available in terms of the parallel usage of both *Bacillus licheniformis* DSM 28710 and salinomycin in poultry diets. From a practical point of view, evaluating the potential interactions between this species and agent is crucial to assess their parallel usage, and the current study confirmed the positive effect of their mixture on the modulation of pH value in the crop and ceca, as well as the GIT microbiota, especially in the jejunum and ceca. Additionally, the results obtained in this study show positive effects of *B. licheniformis* on the growth performance, as well as the influence of both experimental factors used separately in the case of GIT microbiota modulations.

**Abstract:**

The aim of the study was to investigate the effect of *Bacillus licheniformis* and salinomycin supplementation in broiler diets as individual factors or in combination on the growth performance, GIT morphometry, and microbiota populations. Four hundred one-day-old Ross 308 chicks were randomly distributed to four dietary treatments (10 replicates, 10 birds each). The following treatments were applied: NC—no additives; NC + SAL—salinomycin addition (60 mg/kg diet), NC + PRO—*B. licheniformis* DSM 28710 preparation (1.6 × 10^9^ CFU/kg; 500 mg/kg diet), and NC + SAL + PRO—combination of salinomycin and *B. licheniformis*. Probiotic administration resulted in improvement (*p* < 0.05) of the performance parameters, including body weight gain (1–10 d, and 11–22 d) and feed conversion ratio (11–22 d, 1–36 d). An interaction (*p* < 0.05) between experimental factors was observed in terms of lower pH values in the crop (tendency, *p* = 0.053) and ceca. Both factors lowered the alpha diversity and Enterobacteriaceae and promoted Bacillaceae communities in the jejunum (*p* < 0.05). Interactions were also observed in terms of reducing Clostridiaceae in the ceca. In conclusion, the combined use of *B. licheniformis* and salinomycin in broilers’ diets had beneficial effects.

## 1. Introduction

Probiotic preparations used in animal nutrition are among the most commonly implemented tools to enhance growth, maintain intestinal integrity, and improve the overall health status of birds in intensive production conditions [1,2]. The dominant bacteria in the probiotic products belong to *Lactococcus*, *Lactobacillus*, *Bifidobacterium*, *Enterococcus*, *Streptococcus*, and *Bacillus* spp. [3]. Their mode of action in the bird’s gastrointestinal tract is well described, such as modulating the microbial populations, stabilizing microbial homeostasis, adhering to the intestinal mucosa, competitively excluding potentially pathogenic bacteria, and secreting active metabolites (volatile fatty acids, hydrogen peroxide, diacetyl, defensins, and bacteriocins) [4,5,6]. However, indirect effects, such as lowering of the gastrointestinal tract (GIT) environment pH and stimulating the immunological system, are crucial to the favorable probiotic impact. The *Bacillus* genus, including *B. subtilis*, *B coagulans*, *B. amyloliquefaciens*, and *B. licheniformis*, is frequently and successfully examined as a feed additive in poultry diets [7,8,9,10]. *B. licheniformis* is a microorganism that is “generally recognized as safe” and used to prevent the harmful effect of *Clostridium perfringens*, which causes necrotic enteritis in poultry flocks [11]. Furthermore, its positive effects on growth performance parameters and nutrient utilization by microbial enzyme secretion have been observed [12,13,14]. In the available literature, probiotic preparations that include *B. licheniformis* are considered as alternatives to antibiotics or natural growth promoters; nevertheless, there is scarce information about the relation between the commonly used coccidiostats and probiotic bacterial strains that are implemented simultaneously in broiler chicken diets. Salinomycin is globally used as an agent to prevent coccidiosis in poultry production; however, it has a strong ability to modulate the birds’ GIT microbial populations [15]. Its activity based on transporting ions (K^+^ and Na^+^) and disrupting the cell membrane ion gradient is mainly directed against Gram-positive microorganisms [16,17]. The combination of salinomycin and other compounds such as antimicrobials, bacteriocins, probiotics, prebiotics, butyrate, and comparisons of these materials have been repeatedly examined, primarily for the control of coccidiosis [18,19,20,21,22]. However, limited information is available about the relation between salinomycin and probiotic preparations, especially *B. licheniformis*. Therefore, the present study aimed to investigate the effect of *B. licheniformis* and salinomycin used as individual factors or in combination in broiler chicken diets on the growth performance parameters, selected GIT morphometry, and microbiota populations.

## 2. Materials and Methods

### 2.1. Ethics Statement

According to Polish law and the EU directive (no 2010/63/EU), the experiment conducted within this study does not require the approval of the Local Ethical Committee for Experiments on Animals in Poznań. However, all activities complied with the guidelines of the Local Ethics Commission of the Poznań University of Life Sciences (Poznań, Poland) with respect to animal experimentation and care of the animals under the study.

### 2.2. Birds and Housing

A total of 400 one-day-old female Ross 308 chicks obtained from a commercial hatchery were randomly distributed to four dietary treatments, with 10 replicate pens and 10 birds per pen. The experiment was carried out to investigate the growth performance and GIT microbiome community in birds fed diets supplemented with salinomycin or single-strain probiotic product, i.e., *Bacillus licheniformis* (DSM 28710) preparations (powder form), individually or in combination. The birds were kept in floor pens (1.00 × 1.00 m; straw litter) arranged randomly over 36 d. Stock density was established at 10 birds/m^2^. To simulate intensive production conditions, the experimental pens were surrounded by a chicken flock (9000 birds) composed of the birds of the same origin as those used in the trial. All pens were enriched on the same numbers of nipple drinkers and feed hoppers. The chicken house was equipped with artificial, programmable lights (fluorescent), automatic electric heating and forces ventilation. The temperature inside the building was 32–33 °C at the beginning of the test and was reduced by 2–3 °C each week. From the 28th day, the temperature was set at 21 °C, and at the end of the test, it was approximately 18 °C. The rearing conditions were set up to align with the AVIAGEN guidelines [23].

### 2.3. Diets and Feeding Program

The composition of the experimental basal diets is shown in Table 1. Birds had ad libitum access to water and feed for 36 d. The diets used in the present study were calculated to meet or exceed the National Research Council (NRC) nutrient requirements for broilers [24]. The viscous cereals (wheat), animal fat (pig lard), as well as fish meal, were used to provoke intestinal colonization by *Clostridium perfringens* [25,26,27]. The mash diets were prepared using a disc mill (Skiold A/S, Denmark) at 2.5 mm disc distance, mixed without heat treatment at horizontal double band mixer with roller mills (Zuptor, Gostyń, Poland). The diets were produced in the Piast Pasze feed mill (Lewkowiec, Poland) according to ISO 9001:2008 procedures. Starter diets were offered to all birds from 1 to 10 d of age, growers from 11 to 22 d, and finishers from 23 to 36 d of age. No other feed additives were used in the study, such as exogenous enzymes, antioxidants, etc. The following treatments were applied: NC—no additives; NC + SAL—salinomycin addition (60 mg/kg diet), NC + PRO—*B. licheniformis* preparation (1.6 × 10^9^ CFU/kg; 500 mg/kg diet), and NC + SAL + PRO ×combination of salinomycin (60 mg/kg diet) and *B. licheniformis* (1.6 × 10^9^ CFU/kg; 500 mg/kg diet).

### 2.4. Bacillus licheniformis and Salinomycin Preparation

The *B. licheniformis* (DSM 28710) preparation containing viable spores is recognized as safe by the European Food Safety Authorities (ESFA) and approved as a feed additive for use in poultry nutrition by the European Commission (UE, 2017/1904) [28]. The dosage of the probiotic was established according to law and producer recommendations, i.e., at the level of 1.6 × 10^9^ CFU/kg of diet (500 mg/kg of diet). Both the probiotic preparation and ionophore coccidiostat (salinomycin sodium, 60 mg/kg diet; Sacox) were manufactured by Biovet Join Stock Company (Peshtera, Bulgaria). 

### 2.5. Data and Sample Collection

The following variables were measured: body weight (BW); feed intake (FI) on d 10, 22, and 36, and the following was also calculated: body weight gain (BWG) and feed conversion ratio (FCR). The abovementioned traits were obtained at the laboratory scale (NVL6101, OHAUS, Switzerland) with accuracy ±1. At the end of the experiment (36 d), one randomly chosen bird from each replicate (10 birds per treatment) was sacrificed and eviscerated to collect the crop, jejunal, and cecal digesta. The pH value of their content was measured immediately after slaughter using a combined glass and reference electrode (VWR International, pH 1000 L, Leuven, Belgium). The remaining portion of the crop, jejunal, cecal content was gently squeezed directly into flexigrip bags, pooled based on two birds per bag (*n* = 5) and immediately frozen and stored at −80 °C for the next-generation sequencing (NGS) analysis. The jejunum segment was considered to begin at the end of the duodenum and end at Meckel’s diverticulum. The ileum was defined as the small intestinal segment caudal to Meckel’s diverticulum. The selected GIT segment (i.e., duodenum, jejunum, ileum, and ceca) weights were in relation to BW (% BW) and lengths were in relation to BW (cm/kg BW), and they were measured using a laboratory scale PS 600/C/2 (Radwag, Radom, Poland) and linear scale, respectively, after rinsing in distilled water and draining (10 birds per treatment; *n* = 10). 

### 2.6. Bacterial DNA Extraction and Amplification

Total DNA was extracted from 300 ± 10 mg of the crop, jejunal, and cecal digesta samples pooled by segment from two individual birds (n = 5) using a QIAamp Fast DNA stool mini kit (Qiagen, Hilden, Germany) accordingly to the manufacturer’s protocol. First, digesta samples were mechanically lysed using a FastPrep-24 (MP Biomedicals, Santa Ana, CA, USA) and Lysing Matrix A containing garnet matrix and one 1/4" ceramic sphere (MP Biomedicals, Santa Ana, CA, USA). Bacterial DNA presence was detected using Real-Time PCR on a thermocycler Mx3000P (Stratagene, USA) with SYBR Green as the fluorochrome. In the reaction for amplifying 16S rDNA, the following universal reaction primers were used: 1055F 5′-ATGGCTGTCGTCAGCT-3′ and 1392R 5′-ACGGGCGGTGTGTAC-3′. The temperature program of reaction was set as follows: (i) 3 min at 95 °C; (ii) 15 s at 95 °C; (iii) 30 s at 58 °C; 30 s at 72 °C; and (iv) Tm 65 °C to 95 °C. Bacterial DNA was quantified using a Microvolume UV-Vis Spectrophotometer (NanoDrop™ One, Thermo Fisher Scientific, Waltham, Massachusetts, USA) and standardized at 5 ng/μL.

### 2.7. 16 SrDNA Sequencing

The 16SrDNA sequencing analysis was conducted by the GENOMED S.A. (Warsaw, Poland). Briefly, the diversity of microbiota was determined by sequencing the amplified V3-V4 region of the 16S rRNA gene by using the primers 16S Amplicon PCR Forward Primer 5′ TCGTCGGCAGCGTCAGATGTGTATAAGAGACAGCCTACGGGNGGCWGCAG and 16S Amplicon PCR Reverse Primer 5′ GTCTCGTGGGCTCGGAGATGTGTATAAGAGACAGGACTACHVGGGTATCTAATCC. The conditions to the amplification were set up as follows: 3 min at 95 °C; 30 s at 95 °C (25 cycles), 30 s at 55 °C, 30 s at 72 °C, 5 min at 72 °C, and then held at 4 °C. The expected size on a Bioanalyzer trace after the Amplicon PCR step was ≈ 550 bp. The PCR products were cleaned using AMPure XP beads. The libraries were sequenced by running 2 × 300 bp paired-end reads. The PCR products were cleaned, and the library was combined with the sequencing adapters and dual indices using the Nextera XT Index Kit (Illumina, San Diego, CA, USA) according to the 16S Metagenomic Sequencing Library Preparation instruction (Illumina, San Diego, CA, USA). The PCR assay with Nextera XT Index Primers conditions was set up as follows: 3 min at 95 °C; 30 s at 95 °C (eight cycles), 30 s at 55 °C, 30 s at 72 °C, 5 min at 72 °C, and held at 4 °C. Next, for purification of the PCR products AMPure XP beads were used. The library was valid to the expected size on a Bioanalyzer for the final library of ≈630 bp. The libraries were quantified using a fluorometric quantification method using dsDNA binding dyes. Individual concentrations of the DNA libraries were calculated in nM based on the size of the DNA amplicons as determined by an Agilent 2100 Bioanalyzer (Agilent Technologies, Santa Clara, CA, USA).

For sequencing, the individual libraries were diluted to 4 nM, denatured with 10 mM Tris pH 8.5 and spiked with 20% (*v*/*v*) of PhiX. An aliquot of 5 μL of diluted DNA was mixed for pooling the library preparations for MiSeq (Illumina, San Diego, CA, USA) runs. The sample reads were performed >100,000.

### 2.8. Metagenomic Analysis

The microbiome sequences were classified according to the V3 and V4 amplicons and analyzed using a database of 16S rRNA data. Specific sequences 341F and 785R were used for the amplification and library preparation. PCR reactions were performed with Q5 Hot Start High-Fidelity 2X Master Mix, and the reaction conditions were in accordance with the manufacturer’s requirements. Sequencing took place on the MiSeq sequencer using paired-end (PE) technology, 2 × 250 nt, and an Illumina v2 kit. Automatic initial data analyses were performed on the MiSeq apparatus using the MiSeq Reporter (MSR) v2.6 software (Illumina, San Diego, CA, USA). The analysis consisted of two stages: automatic demultiplexing of samples and generating fastq files containing raw reads. The output of sequencing was a classification of reads at several taxonomic levels: kingdom, phylum, class, order, family, genus, and species. Quality analysis of the sequence was conducted with quality control and filtration to obtain high-quality sequences. Valid sequences were screened from samples according to the barcode at both ends of the sequence and corrected for the direction by the primer sequences. All valid and filtered sequences were clustered into Operational Taxonomic Units (OTUs) based on 97% identity. The obtained sequences were BLAST searched against the Greengenes database (greengenes.lbl.gov) to determine the phylogeny of the OTUs. The results were classified at several taxonomic levels: kingdom, phylum, class, order, family, genus, and species. The relative abundance profiles of the cecal microbiota were established according to the OTU abundance of different groups. 

A bioinformatics analysis was performed for the classification of reads by species level using the QIIME software package based on the GreenGenes v13_8 reference sequence database [29,30]. The analysis consisted of the following stages: (i) removal of adapter sequences using the cutadapt program; (ii) quality analysis of reads and removal of low-quality sequences (quality < 20, minimum length 30) using the cutadapt program [31]; (iii) paired sequence connection using the fastq-join algorithm (code.google.com/p/ea-utils); (iv) clustering based on the selected base of reference sequences using the uclust algorithm [32]; (v) chimer sequence removal using the ChimeraSlayer algorithm [33]; and (vi) assigning taxonomy to a selected base of reference sequences using the uclust algorithm [29,32].

### 2.9. Statistical Analysis

A completely randomized design was applied in the study. The Shapiro–Wilk test was used to determine normal distribution. Next, Bartlett’s test was adopted to evaluate the homogeneity of variances. Duncan’s multiple range post-hoc test was used to determine the significance of differences means between treatments at the significance level of *p* < 0.05. Due to the occurrence of non-normality distributed data, Dunn’s test (correction to control the experiment wise error rate —Benjamini–Hochberg) for multiple comparisons followed by a significant Kruskal–Wallis test or Scheirer–Ray–Hare test were used. The analyses were performed via RStudio (v. 1.2.5033; 2009-2019 RStudio, Inc., Boston, USA) using the following packages, i.e., *stats* (v. 3.6.2) [34], *agricolae* (v. 1.3-2) [35], *psych* (v. 1.9.12.31) [36], *dplyr* (v. 0.8.4) [37], *FSA* (v. 0.8.30) [38], *rcompanion* (v. 2.3.25) [39] packages, while charts were generated using *ggplot2* (v. 3.2.1) [40], as well as *ggbiplot* (v. 0.55) packages. 

In the experiment, the following model was implemented:

Y_ij_ = μ + α_i_ + β_j_ + (αβ)_ij_ + δ_ij_,

where Y_ij_ was the observed dependent variable, μ was the overall mean, α_i_ was the effect of salinomycin, β_j_ was the effect *B. licheniformis*, (αβ)_ij_ was the interaction between salinomycin and *B. licheniformis*, and δ_ij_ was the random error.

In terms of the growth performance parameters the replicate pen was used as an experimental unit (*n* = 10); in the case of morphometrical GIT measurements and determining of pH values the 10 birds randomly chosen from each pen was defined as an exp. unit (*n* = 10); the microbiota analyses were done using 10 randomly chosen birds from each experimental pen and digesta samples were pooled by segment from two individual chickens (*n* = 5).

## 3. Results

### 3.1. Birds’ Performance

The effect of *B. licheniform* is addition alone or in combination with salinomycin on the growth performance parameters is shown in Table 2. No interaction (*p* > 0.05) between experimental factors was noticed in terms of BWG, FI, or FCR in each rearing period. However, the main effect of *B. licheniformis* supplementation was increasing the BWG, which was observed in the starter (1–10 d; *p* = 0.016) and grower (11–22 d; *p* = 0.018) period. Moreover, *B. licheniformis* addition reduced the FCR value on d 11–22 (*p* < 0.001) as well as during the entire experimental period (1–36 d; *p* = 0.004). Salinomycin did not have any effect (*p* > 0.05) on the growth performance parameters.

### 3.2. Morphometric Measurements

No effect (*p* > 0.05) of both factors, i.e., *B. licheniformis* and salinomycin addition, separately or as a mixture to the broiler chicken diets was observed on the weights and lengths of selected GIT segment. Based on the abovementioned reason, the authors decided to show the results in Supplementary Table S1.

### 3.3. pH Value of Digesta

A significant interaction between *B. licheniformis* and salinomycin was observed in terms of pH value in the cecal digesta (*p* = 0.046), and a similar tendency (*p* = 0.053) was observed in the crop (Table 3). Salinomycin increased (*p* = 0.001) the pH of the crop digesta, which was not observed with the supplementation of *B. licheniformis* in the broiler diets, which significantly reduced its value (*p* = 0.005). Decreasing (*p* = 0.015) pH was noticed in the cecal content after *B. licheniformis* addition. 

### 3.4. Qualitative Determination of the GIT Microbiota

The NGS analyses were performed using 60 samples to generate a total of 7,228,982 raw sequence reads. After passing the quality filter, there were 7,021,756 (97.13%) sequences. A relative abundance of bacteria was recorded in all experimental groups (99.10%–99.89%).

**Figure 1:**
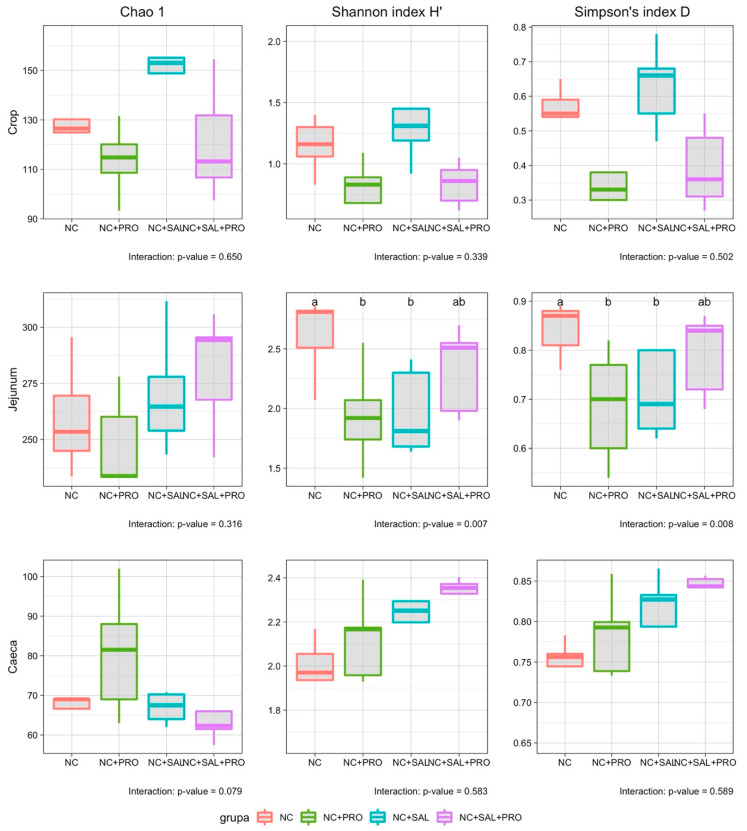
Effect of dietary supplementation of *B. licheniformis* alone or in combination with salinomycin on selected ecological indices of the gastrointestinal tract (GIT) microbiota in the crop, jejunum, and ceca of broiler chickens; means not sharing a common superscript differ significantly (*p* < 0.05); NC—control diet with no additives; NC + PRO—*B. licheniformis* preparation (1.6 × 10^9^ CFU/kg diet); NC + SAL—salinomycin addition (60 ppm); NC + SAL + PRO—a mixture of salinomycin (60 ppm) and *B. licheniformis* (1.6 × 10^9^ CFU/kg diet); means represent 10 pens of one chick each pooled by two (*n* = 5).

In terms of ecological indices, the interaction between experimental factors was observed only in the jejunum in the case of Shannon and Simpson indices (*p* = 0.007, *p* = 0.008, respectively; Figure 1). The NC + SAL as well as NC + PRO treatments decreased the biodiversity in comparison to the control group (NC; *p* = 0.033). However, simultaneous usage of both substances enhanced both, i.e., the Shannon and Simpson indices increased to the NC level (Table S2). The significant effects of *B. licheniformis* were noticed in the crop segment, where the Chao1, Shannon, and Simpsons indices were decreased as an effect of its supplementation (*p* < 0.05). In addition, the salinomycin effect was mainly observed in the cecal digesta, where the Shannon (*p* = 0.007) and Simpsons’ (*p* = 0.015) indices were increased (Table S2).

**Figure 2:**
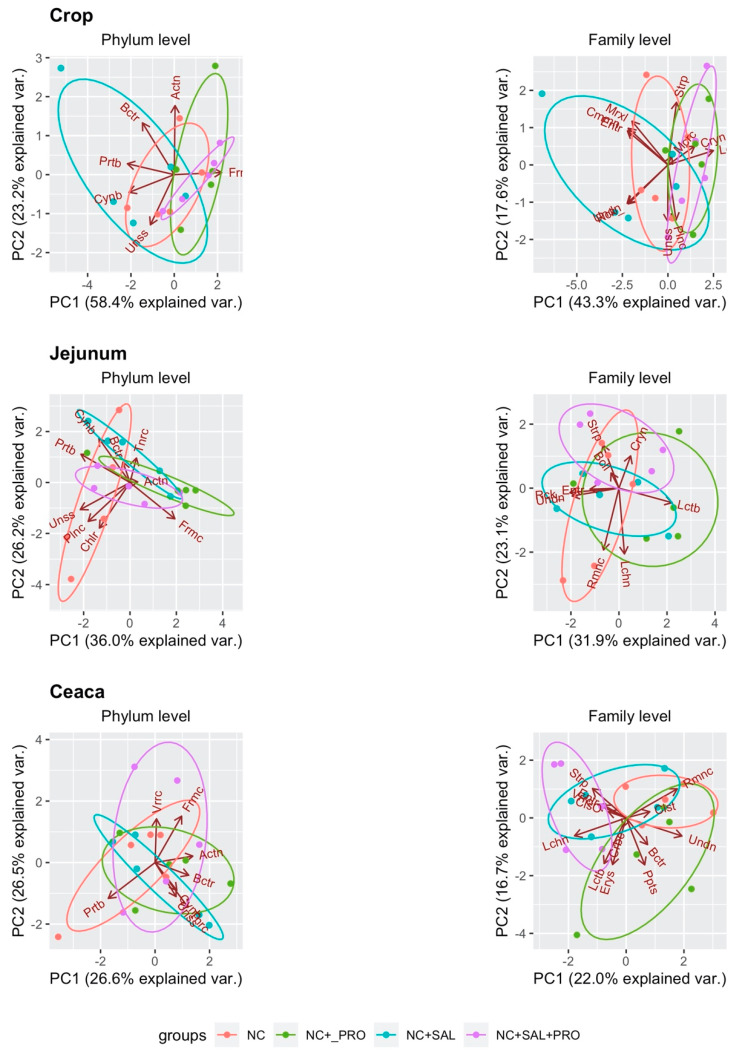
Principal component analysis (PCA) plot of the obtained sequence from the crop, jejunal, and cecal digesta samples: NC—control diet with no additives; NC + PRO—*B. licheniformis* preparation (1.6 × 10^9^ CFU/kg diet); NC + SAL—salinomycin addition (60 ppm); NC + SAL + PRO—a mixture of salinomycin (60 ppm) and *B. licheniformis* (1.6 × 10^9^ CFU/kg diet); means represent 10 pens of one chick each pooled by two (*n* = 5).

To better visualize the beta-diversity, principal component analysis (PCA) was used (Figure 2). The biplot containing PC1 and PC2 showed the distinct clustering of selected microbiota populations in each treatment. The PCA highlighted the disparity between treatments containing the *B. licheniformis* probiotic (NC + PRO and NC + SAL + PRO) and NC as well as the NC + SAL treatment mainly in the crop section. 

**Figure 3:**
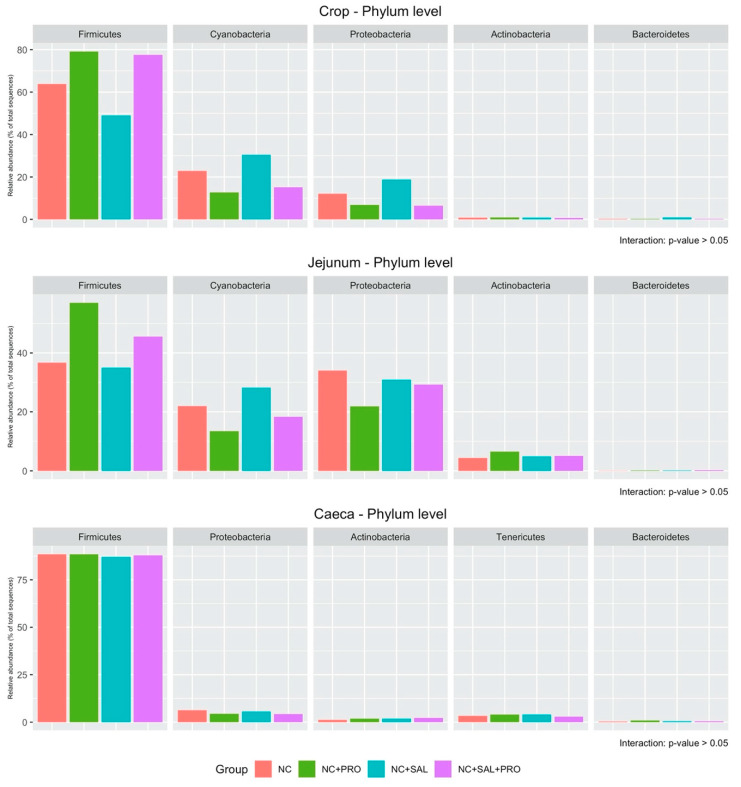
Relative abundance of dominant microbiota at the phylum level in the crop, jejunal, and cecal digesta samples: NC—control diet with no additives; NC + PRO—*B. licheniformis* preparation (1.6 × 10^9^ CFU/kg diet); NC + SAL—salinomycin addition (60 ppm); NC + SAL + PRO—a mixture of salinomycin (60 ppm) and *B. licheniformis* (1.6 × 10^9^ CFU/kg diet); means represent 10 pens of one chick each pooled by two (*n* = 5).

**Figure 4:**
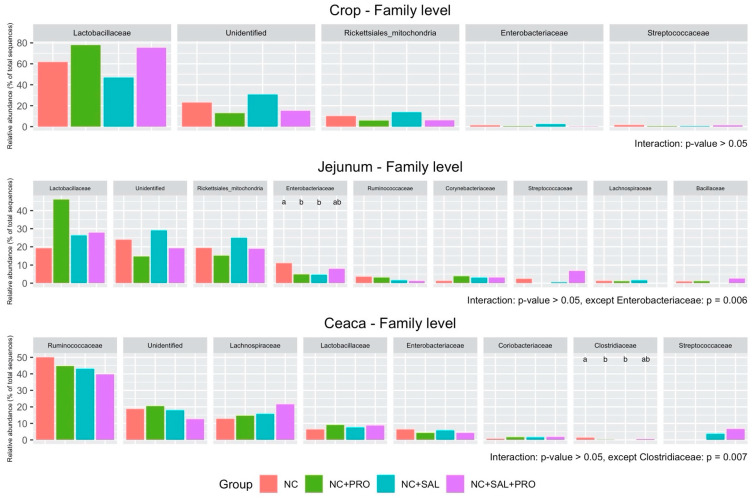
Relative abundance of dominant microbiota at the family level in the crop, jejunal, and cecal digesta samples: NC—control diet with no additives; NC + PRO—*B. licheniformis* preparation (1.6 × 10^9^ CFU/kg diet); NC + SAL—salinomycin addition (60 ppm); NC + SAL + PRO—a mixture of salinomycin (60 ppm) and *B. licheniformis* (1.6 × 10^9^ CFU/kg diet); means represent 10 pens of one chick each pooled by two (*n* = 5).

The relative abundance of dominant microbiota at the phylum level in the crop, jejunum, and ceca is shown in Figure 3. No interaction between experimental factors was observed in each segment (*p* > 0.05). However, the strong effect of *B. licheniformis* was noticed in terms of increasing Firmicutes (*p* = 0.002) and decreasing Cyanobacteria (*p* = 0.002) and Proteobacteria (*p* = 0.002; Table S3) in the crop. Simultaneously, *B. licheniformis* affected Cyanobacteria (*p* = 0.016) by reducing its population in the jejunum segment (Table S4). In the case of cecal content microbiota, only Actinobacteria was enhanced by both experimental factors (*p* < 0.05; Table S5). At the family level, the interaction between *B. licheniformis* and salinomycin was noticed in the case of Enterobacteriaceae (*p* = 0.006) in the jejunum as well as Clostridiaceae (*p* = 0.007) in the ceca (Figure 4). Additionally, an interaction tendency was also observed in the scope of Bacillaceae (*p* = 0.051). Bacteria classified as Enterobacteriaceae (jejunum) were reduced by both experimental factors used separately (*p* = 0.024), whereas Bacillaceae was promoted (*p* = 0.039) by the mixture of both factors (Table S7). The usage of their mixture led to the establishment of Enterobacteriaceae at the same level as the NC. A similar relation was observed in the case of Clostridiaceaea in the cecal samples (Table S8). Supplementation of *B. licheniformis* in the broiler chicken diets increased Lactobacillaceae (*p* = 0.002) and decreased Rickettsiales (*p* = 0.002) as well as Enterobacteriaceae (*p* = 0.005) in the crop (Table S6). In the jejunum segment, only Bacillaceae (*p* = 0.027) was enhanced and unidentified bacteria populations were reduced (*p* = 0.016) by *B. licheniformis*. In the cecal content, salinomycin had a main effect on increasing Strerptococcaceae (*p* = 0.001) as well as Lachnospiraceae and decreasing Ruminococcaceae (*p* = 0.049).

## 4. Discussion

It is well-documented that selected *Bacillus* strains are used in probiotic preparations for broiler chicken diets because of their favorable growth performance, especially final body weight, BWG, and FCR [10,41]. The beneficial role of *B. licheniformis* on the performance parameters was shown in poultry nutrition, including broiler chickens [42], laying hens [43], as well as turkeys [44]. The present study confirmed the valuable effect of *B. licheniformis* DSM 28710 on the BWG as well as FCR. However, in terms of the bird’s performance, no interaction between experimental factors was observed (*p* > 0.05). It should be highlighted that in the available literature, most data compare the effectiveness of probiotic preparations as an antibiotic substituent. However, ionophores, such as salinomycin, monensin, and narasin, are commonly used coccidiostats that have a significant impact on the GIT microbiota [45,46]. Thus, the effect of synchronous usage of coccidiostats as well as probiotics are crucial to understanding their mode of action. Pereira et al. [47] have shown similar effects of *B. subtilis* strain C-3102 and antibiotics, i.e., bacitracin methylene disalicylate and neomycin sulphate used in parallel. The probiotic preparation as well as antibiotics separately resulted in beneficial growth performance results; however, no interaction was observed between them. 

It is well-known that improving bird performance is strictly related to modulating the GIT microbiota [48] via feed additive supplementation, including the ionophore coccidiostat salinomycin [45] as well as *B. licheniformis* [13]. The pH value in the crop, which is the first barrier for potentially pathogenic bacteria [49], was positively reduced by the addition of *B. licheniformis*, thus establishing a friendly environment for the proliferation of Lactobacillaceae populations as the dominant community in this segment [50]. The opposite effect was noted in the case of salinomycin supplementation in the broiler diets. However, the tendency (*p* = 0.053) for interactions between experimental factors indicates that the usage of both additives can maintain beneficial results associated with decreasing pH values below 5.0, which is crucial to limiting Enterobacteriaceae growth in the crop [49]. Thus, it is not surprising that the diversity indices were lower after probiotic supplementation because of increases in the proliferation of Firmicutes (phylum level) and Lactobacillaceae (family level) in this segment. The strong effect of *B. licheniformis* on limiting the growth of Cyanobacteria, Proteobacteria (Enterobacteriaceae, Rickettsiales), and other microbes (Unidentified) was noticed as a result of the inadequate environmental conditions formed in the crop content as well as competitive exclusion. This finding was consistent with Chambers and Gong [51], who mentioned that the probiotic preparations could be efficient agents against Enterobacteriaceae members via enhanced fermentation. 

Similarly, the microbiota in the jejunum was positively changed via *B. licheniformis* supplementation based on limitations in Cyanobacteria as well as unidentified (family level) bacteria. Both salinomycin and *B. licheniformis* reduced the Enterobacteriaceae population when applied separately in the broiler chicken diets. Their simultaneous usage has a similar effect. Furthermore, the mixture of these feed additives boosted Bacillaceae proliferation in this segment. It is well-documented that salinomycin reduces lactic acid fermentation through the limitation of *Lactobacillus/Enterococcus* in the GIT [21]. However, in the present study, no changes in the jejunum pH value, as an indicator of microbial fermentation, were noticed, which was similar to the lack of significant effects on the Lactobacillaceae population. In general, the results of this study were in agreement with those reported by Xu et al. [52], where the following populations were mentioned as predominant in the jejunum: Firmicutes, Proteobacteria, Cyanobacteria, Bacteroidetes, and Actinobacteria. Consistent with the results obtained by Li et al. [53], the alpha-diversity indices in the jejunum section were decreased when the experimental factors were used separately. In the terms of microbial ecology in the cecal content, only salinomycin increased the Shannon and Simpson’s indices. Contrary to this result, bacitracin did not affect the cecal digesta alpha-diversity [54], although the time shift of sampling had a significant impact [55]. Wang et al. [56] reported that supplementation of both salinomycin and bacitracin has an effect only on Simpsons’ index in the cecal microecology. Moreover, the lack of any *B. licheniformis* influence is consistent with the results of Ren et al. [57] and Ma et al. [58], who used *Lactobacillus agilis*, *L. salivarius*, and *B. subtilis* DSM 3231. The main microbiota populations, i.e., Firmicutes, Proteobacteria, Actinobacteria, Tenericutes, and Bacteroidetes, were similar to those reported by Józefiak et al. [59] and Pereira et al. [47]. The favorable relation between Firmicutes and Bacteroidetes was noticed in the current study, which could be linked with the improvement of the birds’ growth performance [60]. Moreover, the Actinobacteria population was significantly increased by both experimental factors. Actinobacteria was mainly represented by Coriobacteriaceae members, which are involved in lipid metabolism [61] and may be considered as healthy GIT indicators [62]. Furthermore, salinomycin increased Lachnospiraceae in the ceca relative to other antibiotics, such as enramycin [63]. Chen and Yu [63] reported that *B. licheniformis* fermented products may reduce Lachnospiraceae depending on the dosage. In the current study, no effect of *B. licheniformis* DSM 28710 on this microbiota population was observed. The Lachnospiraceae community is responsible for degrading fibrous material [64]; however, its additional role in starch and nonstarch polysaccharides (NSP) utilization has also been reported [62]. Conversely, salinomycin decreased the Ruminococcaceae community in the ceca. This finding is inconsistent with that of Manoharan [65], who reported the increased abundance of this community after coccidiostat addition. Furthermore, Torok et al. [66] showed that avilamycin positively affected the Ruminococcaceae population. The Ruminococcaceae members produce butyric as well as formic acids, which as short-chain fatty acids play a crucial role in the limitation of pathogenic bacteria proliferation and stimulate the birds’ growth performance [67,68]. However, in the present study, the frequency of the Ruminococcaceae in the ceca (40%–50%) was high in comparison to the results of other authors, i.e., 19% [69]; 7.29%–10.17% [70]; or >35% [71]. Therefore, a decrease in the value of Ruminococcacae cannot be concluded as a negative effect of salinomycin in this case. Both antibiotic therapy and *B. subtilis* used individually or as a mixture in broiler chicken diets does not have any influence on the Streptococcaceae family in the ceca [47]. Hence, in the present study, there was no interaction observed between experimental factors. However, salinomycin significantly increased the Streptococcaceae community (mainly *Streptococcus* spp.) as a member of Lactobacillales recognized as probiotic microbiota [72]. The usage of both experimental factors separately resulted in lower (*p* = 0.014) Clostridiaceae populations in comparison to the NC group, while their mixture resulted in no difference between treatments. It needs to be emphasized that at the species level, no *C. perfringens* were detected in the samples. However, the obtained OTUs for *Clostridium* spp. indicated the need for further analyses to make a more specific conclusion.

## 5. Conclusions

The results of the present study confirmed that the usage of *B. licheniformis* as a feed additive in broiler chicken diets has beneficial effects on growth performance, especially the BWG and FCR. Furthermore, the usage of both experimental factors resulted in significant changes in pH value in the crop and cecal content and modulation of selected microbiota populations through the whole broilers’ GIT. From a practical point of view, significant interactions between salinomycin and *B. licheniformis* in the scope of pH value regulation (crop, ceca) as well as changes in jejunal alpha-diversity and selected microbiota communities indicate positive modulation in the jejunum and ceca. The current study expands knowledge about the alimentary factor interactions and their effect on the microbiota, especially crop which affects the microbial homeostasis maintenance in the lower GIT segments. 

## Figures and Tables

**Table 1:** Composition and nutritive value of the basal diets.

Ingredient (g·kg^−1^)	Diets		
	1–10 d	11–22 d	23–36 d
Wheat	360.8	362.1	356.0
Maize	250.0	250.0	250.0
Rapeseed expeller	-	40.0	80.0
Rapeseeds	40.0	80.0	60.0
Soy meal, 46.8%	264.7	185.4	161.1
Fish meal, 64%	20.0	20.0	20.0
Hemoglobin	5.0	5.9	5.0
Soy oil	21.1	-	-
Pig lard	-	29.4	44.4
^1^ Vitamin-mineral premix	3.0	3.0	3.0
Monocalcium phosphate	16.8	9.3	5.5
Limestone	8.0	6.4	6.4
NaCl	1.1	1.4	1.7
Na2SO4	2.2	1.5	1.2
L-lysine	2.9	2.5	2.4
L-methionine	2.6	2.0	1.9
L-threonine	1.3	0.9	1.4
L-valine	0.5	0.2	-
Calculated nutritive value (g·kg^−1^)			
^2^ AME_N_, kcal·kg^−1^	3010	3150	3230
Crude protein	216.0	200.0	196.0
Crude fat	58.3	85.2	94.2
Crude fiber	27.1	31.9	33.8
Dig. Lys	12.0	10.7	10.3
Dig. Met + Cys	8.9	8.1	7.9
Calcium-total	8.5	7.0	6.5

^1^ Provided the following per kilogram of diet: vitamin A, 11,166 IU; vitamin D_3_, 2500 IU; vitamin E, 80 mg; vitamin K_3_, 2.50 mg; vitamin B_12_, 0.02 mg; vitamin B_9_, 1.17 mg; choline, 379 mg; vitamin B_5_, 12.50 mg; vitamin B_2_, 7.0 mg; vitamin B_3_, 41.67 mg; vitamin B_1_, 2.17 mg; vitamin B_7_, 0.18 mg; vitamin B_6_, 4.0 mg; ethoxyquin (EMQ), 0.09 mg; Mn (MnO_2_), 73 mg; Zn (ZnO), 55 mg; Fe (FeSO_4_), 45 mg; Cu (CuSO_4_), 20 mg; I (CaI_2_O_6_), 0.62 mg; and Se (Na_2_SeO_3_), 0.3 mg. ^2^ Apparent metabolizable energy corrected to zero nitrogen balance.

**Table 2:** Effect of *Bacillus licheniformis* addition alone or in combination with salinomycin on the growth performance of broiler chickens.

Treatment		Performance											
		1–10 d			11–22 d			23–36 d			1–36 d		
Salinomycin	*B. licheniformis*	BWG ^1^, g	FI ^2^, g	FCR ^3^, g:g	BWG, g	FI, g	FCR, g:g	BWG, g	FI, g	FCR, g:g	BWG, g	FI, g	FCR, g:g
-	-	234	314	1.34	700	1006	1.44	1300	2073	1.60	2234	3393	1.52
+	-	230	318	1.39	705	1002	1.42	1330	2108	1.59	2264	3428	1.52
-	+	239	316	1.32	725	1017	1.40	1331	2125	1.60	2295	3458	1.51
+	+	243	319	1.32	715	1008	1.41	1325	2097	1.58	2283	3425	1.50
Model RMSE ^4^		11.47	8.84	0.08	22.88	32.76	0.02	56.47	11.88	0.03	76.61	102.08	<0.01
Model P		0.070	0.553	0.170	0.081	0.786	0.003	0.575	0.476	0.525	0.302	0.572	0.018
Main effects													
Salinomycin													
None		237	315	1.33	713	1012	1.42	1315	2099	1.60	2264	3425	1.51
60 mg/kg		236	319	1.35	710	1005	1.42	1327	2103	1.59	2274	3426	1.51
*B. licheniformis*													
None		232 ^b^	316	1.37	702 ^b^	1004	1.43 ^a^	1315	2090	1.59	2249	3410	1.52 ^a^
1.6 × 10^9^ CFU/kg		241 ^a^	317	1.32	720 ^a^	1013	1.41 ^b^	1328	2111	1.59	2289	3441	1.50 ^b^
*p*-value													
Salinomycin		0.962	0.177	0.455	0.723	0.550	0.565	0.510	0.880	0.146	0.705	0.970	0.205
*B. licheniformis*		0.016	0.658	0.067	0.018	0.423	<0.001	0.466	0.386	0.967	0.100	0.349	0.004
Interaction terms Salinomycin × *B. licheniformis*		0.275	0.859	0.286	0.331	0.837	0.094	0.316	0.194	0.795	0.380	0.295	0.827

^a–b^ Means not sharing a common superscript differ significantly (*p* < 0.05); ^1^ body weight gain; ^2^ feed intake; ^3^ feed conversion ratio; ^4^ root-mean-square error; means represent 10 pens of 10 chick each (10 replicates).

**Table 3:** Effect of *Bacillus licheniformis* addition alone or in combination with salinomycin on the pH value of the crop, jejunal, and cecal digesta.

Treatment		pH		
Salinomycin	*B. licheniformis*	Crop	Jejunum	Ceca
-	-	4.90	5.92	5.67 ^b^
+	-	5.61	5.93	6.03 ^a^
-	+	4.69	5.80	5.60 ^b^
+	+	4.84	5.90	5.44 ^b^
Model RMSE ^1^		0.43	0.16	0.40
Model P		<0.001	0.345	0.020
Main effects				
Salinomycin				
None		4.79 ^b^	5.86	5.63
60 mg/kg		5.21 ^a^	5.92	5.72
*B. licheniformis*				
None		5.23 ^a^	5.93	5.84 ^a^
1.6 × 10^9^ CFU/kg		4.77 ^b^	5.85	5.52 ^b^
*p*-value				
Salinomycin		0.005	0.350	0.494
*B. licheniformis*		0.001	0.176	0.015
Interaction terms Salinomycin × *B. licheniformis*		0.053	0.432	0.046

^a–b^ superscripts indicate significant differences within a column (*p* < 0.05); ^1^ root-mean-square error; means represent 10 pens of one chick each (10 replicates).

## Supplementary Materials

The following are available online at https://www.mdpi.com/2076-2615/10/5/889/s1, Table S1. Effect of *Bacillus licheniformis* addition alone or in combination with salinomycin on the relative weight of selected sections of the gastrointestinal tract of broiler chicken, Table S2. Effect of dietary supplementation of *B. licheniformis* alone or in combination with salinomycin on selected ecological indices of the GIT microbiota in the crop, jejunum, and ceca of broiler chickens, Table S3. Effect of dietary supplementation of *B. licheniformis* alone or in combination with salinomycin on the relative abundance (at the phylum level) of the dominant microbiota populations in the crop of broiler chickens, Table S4. Effect of dietary supplementation of *B. licheniformis* alone or in combination with salinomycin on the relative abundance (at the phylum level) of the dominant microbiota populations in the jejunum of broiler chickens, Table S5. Effect of dietary supplementation of *B. licheniformis* alone or in combination with salinomycin on the relative abundance (at the phylum level) of the dominant microbiota populations in the ceca of broiler chickens, Table S6. Effect of dietary supplementation of *B. licheniformis* alone or in combination with salinomycin on the relative abundance (at the family level) of the dominant microbiota populations in the crop of broiler chickens, Table S7. Effect of dietary supplementation of *B. licheniformis* alone or in combination with salinomycin on the relative abundance (at the family level) of the dominant microbiota populations in the jejunum of broiler chickens, Table S8. Effect of dietary supplementation of *B. licheniformis* alone or in combination with salinomycin on the relative abundance (at the family level) of the dominant microbiota populations in the ceca of broiler chickens.
