# Supplementary material for: Combination of Bacillus licheniformis and Salinomycin: Effect on the Growth Performance and GIT Microbial Populations of Broiler Chickens
Source: Animals (Basel). 2020 May 20;10(5):889. doi: 10.3390/ani10050889 (PMC7278455; doi:10.3390/ani10050889)
Supplement: Supplementary file 1 [file animals-10-00889-s001.pdf]

animals

MDPI

Table S1. Effect of *Bacillus licheniformis* addition alone or in combination with salinomycin on the relative weight of selected sections of the gastrointestinal tract of broiler chicken.

Treatment		Weight, g/kg of BW				Length, cm/kg of BW			
**Salinomycin**	***B. licheniformis***	**Duodenum**	**Jejunum**	**Ileum**	**Ceca**	**Duodenum**	**Jejunum**	**Ileum**	**Ceca**
-	-	0.69	1.27	0.81	0.30	1.47	3.59	3.32	0.69
+	-	0.70	1.32	0.80	0.27	1.45	3.68	3.10	0.70
-	+	0.68	1.27	0.82	0.24	1.39	3.43	3.08	0.66
+	+	0.68	1.27	0.83	0.29	1.48	3.73	3.23	0.73
Model RMSE[1]		0.06	0.12	0.06	0.05	0.15	0.39	0.36	0.08
Model P		0.879	0.724	0.802	0.086	0.583	0.343	0.425	0.353
Main effects									
Salinomycin									
None		0.69	1.27	0.81	0.27	1.43	3.51	3.20	0.67
60 mg/kg		0.69	1.29	0.81	0.28	1.47	3.70	3.17	0.71
B. licheniformis									
None		0.70	1.30	0.80	0.29	1.46	3.63	3.22	0.69
1.6·× 10^9 CFU/kg		0.68	1.27	0.82	0.27	1.43	3.58	3.16	0.69
p-value									
Salinomycin		0.760	0.567	0.767	0.721	0.494	0.122	0.793	0.121
B. licheniformis		0.458	0.441	0.463	0.152	0.533	0.648	0.651	0.962
Interaction terms<br>Salinomycin x *B. licheniformis*		0.913	0.540	0.554	0.340	0.301	0.406	0.119	0.367

[1] Root-mean-square error; means represent 10 pens of 1 chick each (10 replicates).

Table S2. Effect of dietary supplementation of *B. licheniformis* alone or in combination with salinomycin on selected ecological indices of the GIT microbiota in the crop, jejunum, and ceca of broiler chickens

Treatment		Crop			Jejunum			Ceca		
**Salinomycin**	***B. licheniformis***	*Chao 1*	*Shannon index (H′)*	*Simpson′s index D*	*Chao 1*	*Shannon index (H′)*	*Simpson′s index D*	*Chao 1*	*Shannon index (H′)*	*Simpson′s index D*
-	-	131.0	1.15	0.54	259.4	2.61[a]	0.84[a]	67.7	1.95	0.74
+	-	145.8	1.39	0.63	270.3	1.97[b]	0.71[b]	66.9	2.23	0.81
-	+	113.7	0.83	0.37	247.7	1.94[b]	0.69[b]	80.7	2.12	0.79
+	+	120.7	0.84	0.39	281.1	2.33[ab]	0.79[ab]	64.69	2.32	0.83
SEM[1]		4.47	0.08	0.03	5.75	0.1	0.02	2.35	0.05	0.01
Model P		0.008	0.002	<0.001	0.1999	0.033	0.046	0.051	0.023	0.043
Main effects										
Salinomycin										
None		122.3	0.99	0.45	253.5	2.28	0.76	74.21	2.04[b]	0.76[b]
60 mg/kg		133.3	1.11	0.51	275.7	2.15	0.75	65.80	2.27[a]	0.82[a]
B. licheniformis										
None		138.4[a]	1.27[a]	0.583[a]	264.9	2.29	0.78	67.31	2.09	0.774
1.6·× 10^9 CFU/kg		117.2[b]	0.84[b]	0.380[b]	264.4	2.13	0.74	72.70	2.22	0.808
p-value										
Salinomycin		0.211	0.331	0.261	0.060	0.447	0.747	0.054	0.007	0.015
B. licheniformis		0.023	0.003	<0.001	0.965	0.357	0.364	0.202	0.115	0.135
Interaction terms Salinomycin x *B. licheniformis*		0.650	0.339	0.502	0.316	0.007	0.008	0.079	0.583	0.589

[a-b]Means not sharing a common superscript differ significantly ($p < 0.05$); [1] standard error of the mean; means represent 2 birds pooled from 10 randomly chosen from each treatment (5 replicates).

Table S3. Effect of dietary supplementation of *B. licheniformis* alone or in combination with salinomycin on the relative abundance (at the phylum level) of the dominant microbiota populations in the crop of broiler chickens.

Treatment		Crop – phylum level				
**Salinomycin**	***B. licheniformis***	**Firmicutes**	**Cyanobacteria**	**Proteobacteria**	**Actinobacteria**	**Bacteroidetes**
-	-	63.85	22.79	12.12	0.77	0.24
+	-	48.96	30.39	18.74	0.74	0.94
-	+	79.17	12.75	6.77	0.95	0.14
+	+	77.52	15.11	6.42	0.68	0.19
SEM[1]		3.87	2.26	1.59	0.08	0.19
Model P		0.007	0.011	0.007	0.695	0.646
Main effects						
Salinomycin						
None		71.51	17.77	9.44	0.86	0.19
60 mg/kg		63.24	22.75	12.58	0.71	0.56
B. licheniformis						
None		56.40[b]	26.59[a]	15.43[a]	0.76	0.59
1.6·× 10⁹ CFU/kg		78.35[a]	13.93[b]	6.59[b]	0.82	0.16
p-value						
Salinomycin		0.176	0.175	0.212	0.382	0.595
B. licheniformis		0.002	0.002	0.002	0.715	0.305
Interaction terms Salinomycin x *B. licheniformis*		0.273	0.466	0.168	0.482	0.569*

[a-b]Means not sharing a common superscript differ significantly ($p < 0.05$); [1] standard error of the mean; * nonparametric post hoc Dunn's test after the Scheirer-Ray-Hare test; means represent 2 birds pooled from 10 randomly chosen from each treatment (5 replicates).

Table S4. Effect of dietary supplementation of *B. licheniformis* alone or in combination with salinomycin on the relative abundance (at the phylum level) of the dominant microbiota populations in the jejunum of broiler chickens.

**Treatment**		**Jejunum – phylum level**				
**Salinomycin**	***B. licheniformis***	**Firmicutes**	**Cyanobacteria**	**Proteobacteria**	**Actinobacteria**	**Bacteroidetes**
-	-	36.70	21.97	34.03	4.40	0.03
+	-	35.00	28.23	30.89	4.92	0.14
-	+	57.09	13.44	21.87	6.50	0.16
+	+	45.61	18.33	29.23	5.06	0.19
SEM		4.12	2.00	2.44	0.53	0.03
Model P		0.214	0.046	0.361	0.249	0.379
Main effects						
Salinomycin						
None		46.89	17.70	27.95	5.45	0.10
60 mg/kg		40.31	23.28	30.06	4.99	0.17
B. licheniformis						
None		35.85	25.10^{a}	32.46	4.66	0.09
$1.6 \cdot \times 10^{9}$ CFU/kg		51.35	15.89^{b}	25.55	5.78	0.18
p-value						
Salinomycin		0.413	0.121	0.668	0.151	0.326
B. licheniformis		0.066	0.016	0.171	0.650	0.190
Interaction terms<br>Salinomycin x *B. licheniformis*		0.542	0.843	0.292	0.174*	0.541

$^{a-b}$Means not sharing a common superscript differ significantly ($p < 0.05$); [1] standard error of the mean; * nonparametric post hoc Dunn’s test after the Scheirer-Ray-Hare test; means represent 2 birds pooled from 10 randomly chosen from each treatment (5 replicates).

Table S5. Effect of dietary supplementation of *B. licheniformis* alone or in combination with salinomycin on the relative abundance (at the phylum level) of the dominant microbiota populations in the ceca of broiler chickens.

**Treatment**		**Ceca – phylum level**				
**Salinomycin**	***B. licheniformis***	**Firmicutes**	**Proteobacteria**	**Actinobacteria**	**Bacteroidetes**	**Tenericutes**
-	-	88.4	6.33	1.17	0.39	3.33
+	-	87.1	5.58	1.85	0.54	3.99
-	+	88.5	4.38	1.87	0.82	4.02
+	+	87.9	4.28	2.21	0.54	2.85
SEM		0.96	0.35	0.13	0.15	0.35
Model P		0.539	0.436	0.022	0.684	0.615
Main effects						
Salinomycin						
None		88.4	5.35	1.52[b]	0.60	3.68
60 mg/kg		87.5	4.93	2.03[a]	0.54	3.42
B. licheniformis						
None		87.8	5.96	1.51[b]	0.46	3.66
$1.6 \cdot \times 10^9$ CFU/kg		88.2	4.33	2.04[a]	0.68	3.44
p-value						
Salinomycin		0.199	0.733	0.028	0.650	0.723
B. licheniformis		0.821	0.256	0.024	0.364	0.758
Interaction terms Salinomycin x *B. licheniformis*		0.496*	0.426*	0.435	0.496*	0.222

[a-b]Means not sharing a common superscript differ significantly ($p < 0.05$); [1] standard error of the mean; * nonparametric post hoc Dunn's test after the Scheirer-Ray-Hare test; means represent 2 birds pooled from 10 randomly chosen from each treatment (5 replicates).

Table S6. Effect of dietary supplementation of *B. licheniformis* alone or in combination with salinomycin on the relative abundance (at the family level) of the dominant microbiota populations in the crop of broiler chickens.

Treatment		Crop – family level				
**Salinomycin**	***B. licheniformis***	**Lactobacillaceae**	**Unidentified**	**Rickettsiales_mitochondria**	**Enterobacteriaceae**	**Streptococcaceae**
-	-	61.55	22.85	10.00	1.16	1.34
+	-	46.85	30.43	13.63	2.14	0.41
-	+	77.81	12.79	5.76	0.38	0.44
+	+	75.28	15.14	5.90	0.22	1.32
SEM		3.94	2.26	1.06	0.29	0.35
Model P		0.007	0.011	0.010	0.033	0.240
Main effects						
Salinomycin						
None		69.68	17.82	7.88	0.77	0.89
60 mg/kg		61.07	22.78	9.76	1.18	0.86
B. licheniformis						
None		54.20^{b}	26.64^{a}	11.81^{a}	1.65^{a}	0.87
$1.6 \cdot \times 10^{9}$ CFU/kg		76.55^{a}	13.97^{b}	5.83^{b}	0.30^{b}	0.88
p-value						
Salinomycin		0.167	0.176	0.264	0.910	0.176
B. licheniformis		0.002	0.002	0.002	0.005	0.189
Interaction terms<br>Salinomycin x *B. licheniformis*		0.322	0.467	0.299	0.405*	0.417*

$^{a\text{-}b}$Means not sharing a common superscript differ significantly ($p < 0.05$); [1] standard error of the mean; * nonparametric post hoc Dunn's test after the Scheirer-Ray-Hare test; means represent 2 birds pooled from 10 randomly chosen from each treatment (5 replicates).

Table S7. Effect of dietary supplementation of *B. licheniformis* alone or in combination with salinomycin on the relative abundance (at the family level) of the dominant microbiota populations in the jejunum of broiler chickens.

**Treatment**		**Jejunum – family level**								
**Salinomycin**	***B. licheniformis***	**Lactobacillaceae**	**Unidentified**	**Rickettsiales_mitochondria**	**Enterobacteriaceae**	**Ruminococcaceae**	**Corynebacteriaceae**	**Streptococcaceae**	**Lachnospiraceae**	**Bacillaceae**
-	-	19.12	23.81	19.36	11.03[a]	3.54	1.19	2.32	1.23	0.92
+	-	26.20	28.98	24.82	4.50[b]	1.53	2.88	0.41	1.41	0.00
-	+	46.03	14.69	15.03	4.86[b]	3.14	3.84	0.00	1.09	1.10
+	+	27.83	19.20	18.92	7.91[ab]	1.12	3.04	6.72	0.00	2.47
SEM		4.84	2.02	1.98	0.92	0.56	0.75	1.27	0.27	0.32
Model P		0.249	0.058	0.401	0.024	0.361	0.680	0.410	0.169	0.039
Main effects										
Salinomycin										
None		32.58	19.25	17.19	7.948	3.34	2.51	1.16	1.16	1.01
60 mg/kg		27.01	24.09	21.87	6.206	1.33	2.96	3.56	0.70	1.24
B. licheniformis										
None		22.66	26.40[a]	22.09	7.77	2.53	2.03	1.36	1.32	0.46[b]
1.6·× 10^9 CFU/kg		36.93	16.95[b]	16.97	6.39	2.13	3.44	3.36	0.55	1.79[a]
p-value										
Salinomycin		0.558	0.186	0.253	0.263	0.088	0.338	0.256	0.457	0.682
B. licheniformis		0.145	0.016	0.213	0.372	0.720	0.491	0.829	0.186	0.027
Interaction terms<br>Salinomycin x *B. licheniformis*		0.194	0.927	0.845	0.006	0.997	0.730*	0.214*	0.098*	0.051

[a-b]Means not sharing a common superscript differ significantly ($p < 0.05$); [1] standard error of the mean; * nonparametric post hoc Dunn's test after the Scheirer-Ray-Hare test; means represent 2 birds pooled from 10 randomly chosen from each treatment (5 replicates).

Table S8. Effect of dietary supplementation of *B. licheniformis* alone or in combination with salinomycin on the relative abundance (at the family level) of the dominant microbiota populations in the ceca of broiler chickens.

Treatment		Ceca – family level							
**Salinomycin**	***B. licheniformis***	**Ruminococcaceae**	**Unidentified**	**Lachnospiraceae**	**Lactobacillaceae**	**Enterobacteriaceae**	**Coriobacteriaceae**	**Clostridiaceae**	**Streptococcaceae**
-	-	50.0	18.63	12.68	6.29	6.23	0.70	1.25[a]	0.00
+	-	42.9	17.87	15.65	7.35	5.57	1.48	0.00[b]	3.58
-	+	44.6	20.39	14.69	8.97	4.17	1.56	0.19[b]	0.00
+	+	39.7	12.50	21.46	8.69	4.18	1.84	0.54[ab]	6.65
SEM		1.50	1.17	1.20	0.75	1.07	0.22	0.15	0.98
Model P		0.149	0.085	0.045	0.589	0.601	0.311	0.014	0.007
Main effects									
Salinomycin									
None		47.3[a]	19.51	13.69[b]	7.63	5.20	1.09	0.72	0.00[b]
60 mg/kg		41.3[b]	15.19	18.56[a]	8.02	4.87	1.70	0.27	5.19[a]
B. licheniformis									
None		46.5	18.25	14.17	6.82	5.90	1.13	0.62	2.36
1.6·× 10^9 CFU/kg		42.2	16.45	18.07	8.83	4.17	1.66	0.37	2.84
p-value									
Salinomycin		0.049	0.055	0.031	0.804	0.733	0.177	0.105	0.001
B. licheniformis		0.227	0.402	0.075	0.208	0.306	0.231	0.370	0.579
Interaction terms									
Salinomycin x *B. licheniformis*		0.940*	0.108	0.369	0.668	0.404*	0.571	0.007*	0.579*

[a-b]Means not sharing a common superscript differ significantly ($p < 0.05$); [1] standard error of the mean; * nonparametric post hoc Dunn's test after the Scheirer-Ray-Hare test; means represent 2 birds pooled from 10 randomly chosen from each treatment (5 replicates).
